# Correction: The biotoxin BMAA promotes dysfunction via distinct mechanisms in neuroblastoma and glioblastoma cells

**DOI:** 10.1371/journal.pone.0296579

**Published:** 2024-01-02

**Authors:** Bryan Burton, Kate Collins, Jordan Brooks, Karly Marx, Abigail Renner, Kaylei Wilcox, Ellie Moore, Keith Osowski, Jordan Riley, Jarron Rowe, Matthew Pawlus

There are errors in the Funding section. The correct Funding statement is: Research reported in this publication was supported by the South Dakota Biomedical Research Infrastructure Network (SD BRIN) through an Institutional Development Award (IDeA) from the National Institute of General Medical Sciences of the National Institutes of Health under grant number P20GM103443. The content is solely the responsibility of the authors and does not necessarily represent the official views of the National Institutes of Health. Cell culturing and functional assays were performed by WestCore at Black Hills State University. The funders had no role in study design, data collection and analysis, decision to publish, or preparation of the manuscript.
