# Supplementary figures and images for: The biotoxin BMAA promotes dysfunction via distinct mechanisms in neuroblastoma and glioblastoma cells
Source: PLoS One. 2023 Mar 9;18(3):e0278793. doi: 10.1371/journal.pone.0278793 (PMC9997973; doi:10.1371/journal.pone.0278793)

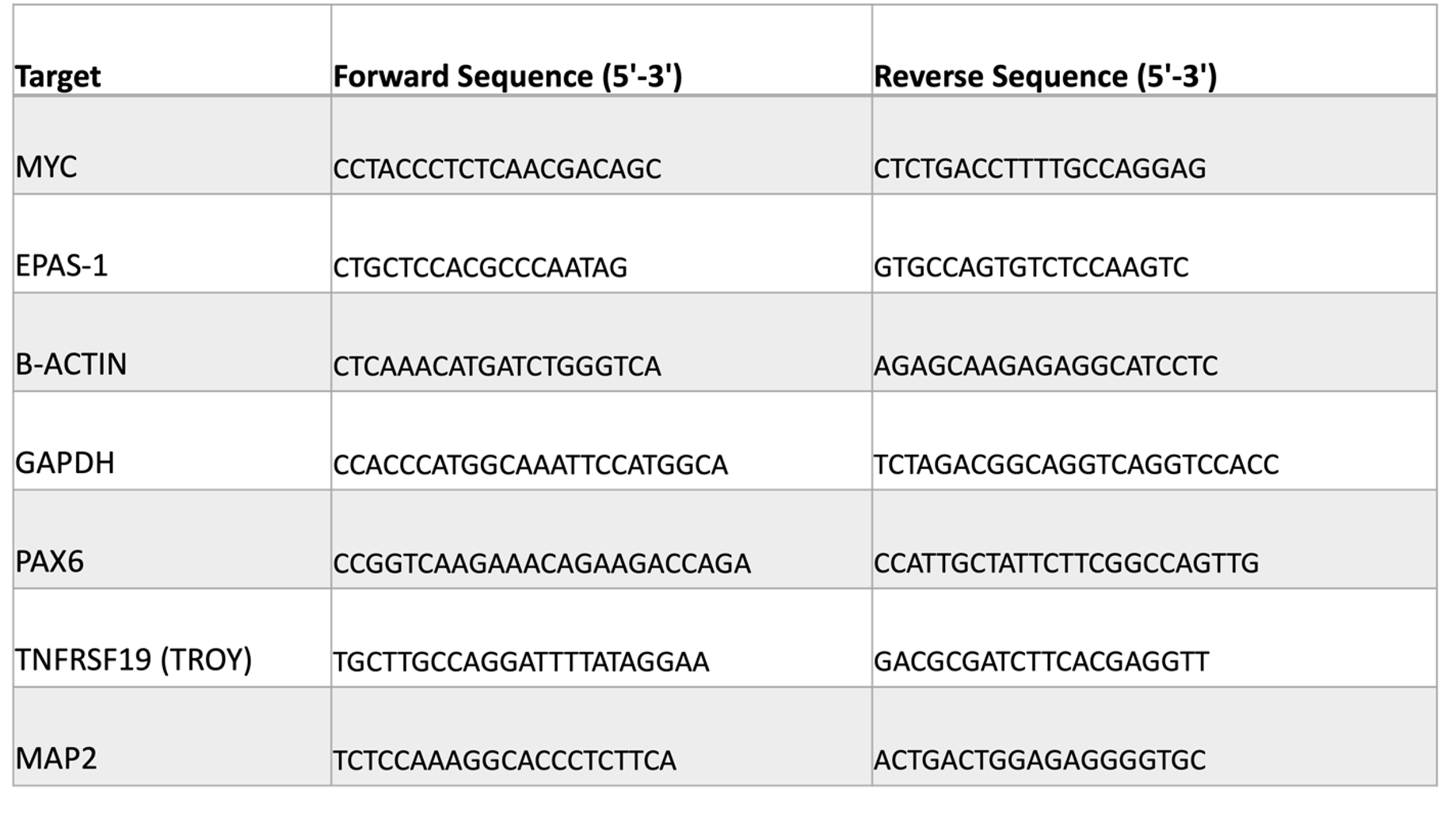

Supplement: S1 Table — All primer sets designed to detect target gene mRNA were validated for their product specificity and amplification efficiency using product electrophoresis, melt curve and standard dilution analysis. Amplification efficiencies of primer sets were between 90 and 110%. (TIF) [file pone.0278793.s001.tif]
